# What Is the Current Role and What Are the Prospects of the Robotic Approach in Liver Surgery?

**DOI:** 10.3390/cancers14174268

**Published:** 2022-08-31

**Authors:** Emre Bozkurt, Jasper P. Sijberden, Mohammed Abu Hilal

**Affiliations:** 1 Department of Surgery, Poliambulanza Foundation Hospital, 25124 Brescia, Italy; 2 Department of Surgery, Hepatopancreatobiliary Surgery Division, Koç University Hospital, Istanbul 34010, Turkey; 3 Department of Surgery, Amsterdam UMC Location University of Amsterdam, Meibergdreef 9, 1105 AZ Amsterdam, The Netherlands; 4 Department of Surgery, University Hospital Southampton NHS Foundation Trust, Southampton SO16 6YD, UK

**Keywords:** liver, surgery, robotic surgery, minimally invasive

## Abstract

**Simple Summary:**

Robotic liver surgery is being applied with increasing frequency. Comparable, and in specific settings superior, perioperative outcomes compared to laparoscopic liver surgery have been reported. In its current form, the most commonly mentioned advantage of robotic surgery is improved dexterity. Important obstacles to its wider implementation in daily clinical practice are the associated costs, technical difficulties, and a scarce amount of evidence. Robotic liver surgery will likely continue to evolve in parallel with technological developments that enhance the robots’ abilities.

**Abstract:**

In parallel with the historical development of minimally invasive surgery, the laparoscopic and robotic approaches are now frequently utilized to perform major abdominal surgical procedures. Nevertheless, the role of the robotic approach in liver surgery is still controversial, and a standardized, safe technique has not been defined yet. This review aims to summarize the currently available evidence and prospects of robotic liver surgery. Minimally invasive liver surgery has been extensively associated with benefits, in terms of less blood loss, and lower complication rates, including liver-specific complications such as clinically relevant bile leakage and post hepatectomy liver failure, when compared to open liver surgery. Furthermore, comparable R0 resection rates to open liver surgery have been reported, thus, demonstrating the safety and oncological efficiency of the minimally invasive approach. However, whether robotic liver surgery has merits over laparoscopic liver surgery is still a matter of debate. In the current literature, robotic liver surgery has mainly been associated with non-inferior outcomes compared to laparoscopy, although it is suggested that the robotic approach has a shorter learning curve, lower conversion rates, and less intraoperative blood loss. Robotic surgical systems offer a more realistic image with integrated 3D systems. In addition, the improved dexterity offered by robotic surgical systems can lead to improved intra and postoperative outcomes. In the future, integrated and improved haptic feedback mechanisms, artificial intelligence, and the introduction of more liver-specific dissectors will likely be implemented, further enhancing the robots’ abilities.

## 1. Introduction

Minimally invasive surgical systems have been continuously evolving due to technological developments, the need to eliminate human error, to facilitate the surgeon in performing procedures that are challenging both by the open and minimally invasive approach, and the continuous need to improve clinical outcomes. The increasing and widening use of the minimally invasive approach has also led to the swift adoption of the robotic approach in major abdominal surgical procedures. The robotic approach has also been adopted in many other fields, for example, breast cancer and reconstruction surgery [1]. Although the effectiveness of robotic surgery has been proven for several indications, its use is still limited due to relatively high costs, technical difficulties, and insufficient strong evidence of its usefulness in challenging procedures such as liver resections [2,3].

Nevertheless, experienced centers have reported several benefits of minimally invasive liver surgery (MILS) in selected patients. In this context, less postoperative pain, less bleeding, a lower surgical site infection rate and a shorter hospital stay are commonly mentioned advantages. However, whether robotic liver surgery has merits over laparoscopic liver surgery is still very much a matter of debate [4,5,6].

**Figure 1:**
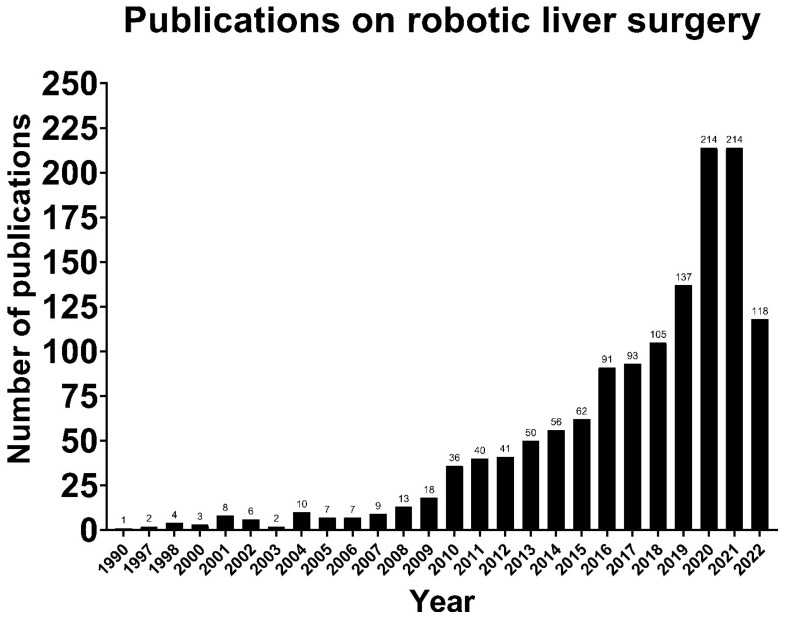
The number of publications on robotic liver surgery increasing over time (Source: PubMed).

Although robotic liver surgery is now widely applied, no standardized, replicable and safe technique has been described yet, despite the increasing literature on the topic (Figure 1). This is possibly due to the specific technical difficulties in liver surgery and a lack of customized surgical instruments, especially for the parenchymal transection phase [7]. The aforementioned example has made some surgeons wary of the application of robotic liver surgery. Nevertheless, the first robotic liver resection was already reported in 2006, and thereafter, the usage of robotic liver surgery has increased rather quickly due to the acquired experience in laparoscopy since the early 1990s [8,9]. In the meantime, an expansion of the indications for robotic liver surgery has taken place, from wedge resections and segmentectomies in the initial phase, to hemi liver resections, extended hemi liver resections, posterosuperior segmentectomies, donor liver resections, and ALLPS in the present day [10,11,12].

Therefore, this review aims to summarize the current literature on robotic liver surgery and gain insight into the potential future wide implementation of this approach.

## 2. Methods

### 2.1. Literature Search Strategy

The references for this narrative review were obtained from Medline, PubMed, the Cochrane Library and Embase database from their inception to June 2022. The following search terms were used: ‘Liver’, ‘Surgery’, ‘Robotic Surgery’, ‘Minimally invasive surgery’, ‘Laparoscopic surgery’, ‘Learning curve’, ‘Operative time’, ‘Blood loss’, ‘Conversion’, ‘Bile leak’, ‘Liver failure’, ‘Length of hospital stay’, ‘Cost effectiveness’, ‘Resection margin’, ‘Complex procedures’, ‘Training in surgery’, ‘Artificial intelligence’ and ‘Machine learning’.

### 2.2. Study Selection

We gave priority to multicenter comparative studies and manuscripts that were conducted by International Study Groups. The reference list of all included articles was searched to identify other potentially relevant studies. Common definitions that were defined by the International Study Group of Liver Surgery were used to define postoperative outcome measurements.

## 3. Learning Curve

The learning curve represents the number of surgical procedures that a surgeon performs to reach a certain level of skill and experience that permits one to perform a procedure safely and efficiently. The pace of this learning process can be affected by many factors including personal experience, previously acquired skills, innate abilities, and the competencies of the surgeon. In addition, the way learning curves are defined varies, and whilst many researchers have looked at the learning curves by measuring operative time, others have considered the conversion rates and the feasibility of the procedure to define a learning curve [13]. Moreover, some authors have suggested that more outcomes should be considered when learning curves are defined, such as complication rates, a return to the operating theatre, and the need for percutaneous drainage [14,15]. All this and the varying complexity of different surgical procedures lead to a pragmatic definition of the learning curves and the need for their careful assessment.

Having said this, the majority of surgeons would agree that surgical success is parallel to the level of experience and the completion of the learning curve for a specific procedure. This success can be defined by measurable improved perioperative outcomes, directly linked to the level of experience [16]. Based on this concept, the learning curve can be divided into two subsequent learning phases, namely, feasibility and proficiency [17]. Several studies in the field of pancreatic surgery have associated the first learning phase with gradually improved intraoperative outcomes, while the last phase has been associated with improved postoperative outcomes [15]. In the field of liver surgery, different learning curve threshold values were defined for several procedures. In a comparative study by Connor et al., the learning curve for robotic minor liver resections was suggested to be completed when a surgeon had performed 25 consecutive cases, linking the cases performed after this threshold with less blood loss and fewer complications [18]. For major liver resections, Chen et al. reported a statistically significantly shorter operation time, less blood loss, and a shorter hospital stay after 52 cases [19]. In the Southampton consensus guidelines, published in 2018, the learning curve for laparoscopic liver surgery was reported as 60 cases for minor resections and an additional 55 cases for major resections [20]. Therefore, the robotic approach seems to have a shorter learning curve. Simulation-based training and the opportunity for a second surgeon to actively participate in the surgical procedure with the second console provided by robotic surgical systems are thought to be the important factors contributing to this faster learning process. Learning curve studies comparing surgeons with no previous experience in both robotic and laparoscopic surgery have also shown that robotic surgery has a shorter learning curve [21]. In studies comparing the suture performance of trainees in laparoscopic and robotic modules, the performance of the robotic surgery group was better, and the robotic performance of expert laparoscopic surgeons was also better than their laparoscopic performance. These findings support the idea that surgeons develop their minimally invasive skills faster with the robotic platform [22].

## 4. Intraoperative Outcomes

### 4.1. Operative Time

Operation time is the time from the induction of anesthesia to wound closure. A longer operation time has frequently been associated with early postoperative complications in the literature, and it has been reported that the duration of surgery may be a modifiable risk factor in the prevention of these complications [23,24,25]. For open liver surgery, a study has found that the rate of pulmonary complications increases as the operation time increases, and a shorter operation time after completion of the learning curve has been associated with better postoperative outcomes in this regard [25]. For laparoscopic liver surgery, Dagher et al., in their study conducted to determine risk factors causing postoperative complications, correlated each one-hour increase in operation time with a 60% increased risk of complications, and they emphasized the importance of a shorter operative time for good outcomes in minimally invasive liver surgery [24].

The reported data on the operative time for robotic liver resections are heterogeneous, mainly because many studies have not made the distinction between minor and major liver resections. The reported mean operative times for robotic major liver resections range from 229.4 to 621 min, and for minor liver resections from 175 to 403 min [26,27,28,29,30,31,32,33,34]. In a comparative study conducted by Yu et al., the operative time for laparoscopic and robotic liver surgery was not significantly different (240.9 ± 68.6 and 291.5 ± 85.1 min, respectively) [35]. In a recently published meta-analysis by Kamarajah et al., robotic liver resections were associated with a significantly longer operative time when compared to laparoscopic liver resections [36]. The major factor that might prolong the operative time of robotic liver resections is the unavailability of the Cavitron Ultrasonic Surgical Aspirator (CUSA). When the CUSA or an equivalent transection technique can be used in robotic liver surgery, the operative time will likely shorten.

### 4.2. Blood Loss

Intraoperative blood loss is one of the commonly used outcome measures to evaluate the intraoperative course, although its measurement methods are controversial. Extensive intraoperative blood loss and a need for the perioperative transfusion of blood products is associated with higher postoperative complication rates and shorter long-term survival [37]. Moreover, the adoption of surgical techniques that reduce the amount of intraoperative bleeding, and advances in surgical technology—especially in minimally invasive surgery, help to limit the amount of blood loss during surgery [38].

In the literature, it is commonly stated that robotic liver surgery is associated with less blood loss when compared to open liver surgery [4,5,39]. Proponents of the robot have suggested that the improved dexterity of the robotic approach would also make it superior, in terms of blood loss, when compared to the laparoscopic approach. There is some evidence that supports this theory, reporting a blood loss of 20 to 100 mL during robotic liver surgery versus 50 to 250 mL in laparoscopic liver surgery [40,41]. Additionally, less intraoperative blood loss was reported in subgroup analyses of complex resections performed with a robotic approach [42].

### 4.3. Conversion

Disease extent, anatomical variations, previous abdominal surgery, technical difficulties, and life-threatening intraoperative incidents are the main reasons for conversion during minimally invasive liver surgery. During minimally invasive surgery, an ‘elective’ (proactive) conversion can be performed as a precautionary measure before any complication develops, or it can be ‘emergent’ (reactive), as a result of an intraoperative unfavorable incident [43]. Elective conversions have been associated with better postoperative outcomes than emergency conversions [44,45]. In a study including the data from liver centers across Europe, in which the relationship between conversions and postoperative outcomes was investigated, emergent conversions were associated with five times higher complication and 90-day mortality rates [46]. In a study comparing conversions in robotic and laparoscopic rectal surgery, Crippa et al. reported that the emergent conversion rate was comparable for robotic and laparoscopic procedures, while the elective conversion rate was lower in the robotic surgery group [47]. This result suggests that a stricter patient selection process is applied for robotic surgery, thereby creating a bias possibly influencing the conversion rates and other perioperative outcomes in studies comparing the laparoscopic and robotic approach.

Nevertheless, robotic surgery has generally been associated with a lower conversion rate, compared to the laparoscopic approach, for various surgical procedures [48,49]. This difference becomes more evident when the volume-weighted conversion rate is calculated [50]. In the development phase of robotic liver surgery, conversion rates as high as 10% were reported. In the recent literature, conversion rates of 5% after completion of the learning curve are reported, which is lower than the conversion rates reported for laparoscopic liver surgery [51,52]. Robotic surgical platforms especially seem to offer a benefit for the resection of lesions in the posterosuperior segments, which are relatively difficult to reach, and major liver resections [27,33]. Aside from this, the fact that major intraoperative complications can be managed more easily with robotic surgery, compared to laparoscopic surgery, supports lower conversion rates [52].

## 5. Postoperative Outcomes

### 5.1. Bile Leak

Bile leakage is one of the most commonly occurring postoperative complications after liver surgery with reported rates up to 8.7%, despite technological developments and current mitigation strategies, and it adversely affects the postoperative course by increasing the rate of intraabdominal infections, liver failure, and increasing the length of stay [53,54]. The type of surgery, parenchymal transection technique, resection type, vessel sealing strategy, intraoperative air leak test, cholangiography, fibrin glue usage and drain usage are the main modifiable factors and techniques that are used for both the diagnosis and prevention of postoperative bile leakage [55,56,57,58].

In a study assessing the occurrence of bile leakage after minimally invasive and open liver surgery, minimally invasive surgery was associated with significantly lower bile leak rates in the overall cohort that included both major and minor resections. However, no statistically significant difference between the two groups was found in the major liver resection subgroup [59]. Patient selection criteria for minimally invasive surgery are thought to be responsible for these results. In a study conducted by Abu Hilal et al. in which 13,379 patients from 15 centers were included, significantly less clinically relevant postoperative bile leakage was detected in the laparoscopic liver resection group compared to open surgery (2.6% vs. 6%) [60].

Additionally, two recently published propensity score matched studies comparing minimally invasive and open liver surgery have also found decreased bile leak rates in the minimally invasive group [60,61]. However, an additional advantage of the robot in this context was not proven, as Kamarajah et al. reported in their meta-analysis that included 12 studies, showing that there was no significant difference in the occurrence of bile leak after robotic and laparoscopic liver resections [36].

### 5.2. Post Hepatectomy Liver Failure

Post hepatectomy liver failure is a serious complication and is defined by the International Study Group of Liver Surgery as a worsening of the liver’s synthetic, excretory and detoxifying functions, which is supported by an elevated INR and bilirubin level after postoperative day five [62]. Its incidence varies according to liver pathology, and it is seen more frequently in primary liver malignancies such as hepatocellular carcinoma and cholangiocarcinoma than in secondary liver malignancies such as colorectal liver metastasis. In addition to the pathology treated, the type of surgery is one of the main factors affecting post hepatectomy liver failure.

Post hepatectomy liver failure rates as high as 13% have been reported, and it is one of the most important causes of mortality after liver resections [63]. Fortunately, the rate of post hepatectomy liver failure has decreased in the current era, especially following the introduction of minimally invasive approaches [64,65]. Zhang et al. even reported zero liver failure following robotic liver resections, versus 0.6% liver failure in the open liver resection group, in their propensity score matched study [4]. In a study by Tee et al. including only elderly patients, the grade B/C liver failure rates were significantly different following open and minimally invasive resections (4.84% versus 0.41%, respectively) [61]. In a nationwide study comparing open, laparoscopic, and robotic liver resections, the robotic group had the lowest liver failure rate (1.1%, 1.1%, and 0.7%, respectively) [66].

### 5.3. Length of Hospital Stay

The length of hospital stay is an outcome that has a direct impact on patients, in terms of physical and social healing, and society, in terms of overall costs. It is now clearly known that one of the most important advantages of minimally invasive surgery is a faster functional recovery, which leads to a shorter length of hospital stay [67,68]. A significantly shorter length of hospital stay was also reported for robotic surgery, compared to open surgery, for minor liver resections in the posterosuperior liver segments by Nota et al. (4 versus 8 days, respectively) [69]. Steward et al. also reported that robotic liver surgery had a shorter hospital stay with 2.2 days versus 6.2 days in their study that compared robotic and open minor liver resections [70]. In a meta-analysis conducted by Qui et al. comparing robotic and laparoscopic liver resections, no statistically significant difference in the length of hospital stay of the two groups could be found [71]. A meta-analysis conducted by Hu et al. confirmed this finding [72].

### 5.4. Cost-Effectiveness

The most important factor limiting the spread of robotic surgery is thought to be cost. Further widespread use of robotic surgery mainly requires a lower cost of robotic systems. Therefore, cost-reducing methods in minimally invasive surgery have always attracted the attention of surgeons and medical device manufacturers.

The clinical advantages provided by minimally invasive surgery, such as lower complication rates, readmission rates, less pain, and a shorter length of hospital stay offer a cost advantage by reducing the costs associated with postoperative patient care by allowing a faster recovery process. Functional recovery and the length of hospital stay are directly related to direct and indirect healthcare costs. Although it is a well-known fact that minimally invasive surgery is associated with higher surgical costs, the total hospital costs are generally lower for patients treated with a minimally invasive approach due to the improved postoperative outcomes [73,74,75]. Specifically for robotic surgery, Wu et al. reported higher perioperative costs with low inpatient care costs [76].

An up-to-4-days shorter length of hospital stay (2.2 vs. 6.2 days) and decreased complication rates after robotic surgery have also been reported in the literature as the main factors decreasing the overall costs in comparison to open surgery [70]. Stewart et al. revealed that the total costs of minor liver resections increased by an additional $2483 when patients had postoperative complications, while readmissions within 1 month resulted in an additional cost of $2516 [70]. This result confirms the cost-reducing effect of the more often uneventful healing process following minimally invasive surgery.

Another factor that is likely to reduce the costs of robotic surgery is the competition in the market that is expected to occur with the launch of newly developed robotic surgical platforms. Aside from this, a greater implementation and wider expansion of robotic liver surgery would increase the experience with this technique, and probably improve its associated perioperative outcomes, likely reducing its associated costs.

### 5.5. Resection Margin

The resection margin is one of the important quantitative postoperative findings after oncologic surgery that is directly associated with long-term outcomes such as recurrence-free and overall survival. Technological advances have allowed minimally invasive surgical techniques to achieve R0 resection rates that are comparable with the traditional open approach. The reported R0 resection rates of robotic resections for colorectal liver metastases range from 73.7 to 100%. A comparative study by Beard et al. assessing the oncological outcomes after robotic and laparoscopic liver resections for colorectal liver metastases reported a 73.7% R0 resection rate in the robotic liver surgery group, which was comparable to the rate of 77.4% in the laparoscopic group [77]. Two recent meta-analyses reported similar results for liver resections performed for all indications [78,79].

Duarte et al. performed a study in which they compared the outcomes following minimally invasive and open right hepatectomies for all indications [80]. While they found comparable R0 resection rates in both groups, wider resection margins were obtained in the minimally invasive group, which may lead to a lower probability of disease recurrence after minimally invasive surgery [80].

In recent publications, histopathological factors and tumor biology were mentioned as the most important predictors of recurrence, and a tumor-free resection margin is deemed sufficient in terms of margin width [81]. It is thought that higher R0 resection rates can be achieved by precise dissection, and robotic surgical systems may facilitate this by integrating radiological imaging with artificial intelligence technology.

### 5.6. Complex Procedures

Resections involving three or more contiguous segments, resections for lesions situated in the posterosuperior segments, resections following modulation of the future liver remnant, two-stage hepatectomies, redo resections or resections requiring a concomitant bilio-enteric anastomosis are deemed complex [82]. The robotic approach might have advantages that are especially useful in these types of situations, such as improved control of the surgical field, bleeding control, and suturing capacity due to the articulation capability of patient-side manipulators. A recently published propensity score-based analysis supports this view, reporting no clinically significant benefits of the robotic approach over the laparoscopic approach for procedures of low- and intermediate difficulty, but merits the use of the robot during technically complex cases in terms of less blood loss and conversions [83]. Despite the advantages of the robots’ improved dexterity during complex surgical procedures, the lack of a specific energy device for the liver transection is an important disadvantage in this setting. For this reason, it may still be necessary to use laparoscopic energy devices or conventional methods, such as the clamp crush technique adapted to robotic arms using force bipolar, during the liver parenchyma transection phase. In addition to this, Croner et al. described the three device (3D) method that enables the exposition of the intrahepatic vascular and biliary tracts using the monopolar scissors and bipolar Maryland forceps of the robot, and a laparoscopic-guided waterjet [84].

Tumor size is another important parameter for resection planning. In parallel with surgical and technological developments, a better understanding of liver anatomy and improvements in preoperative patient preparation have enabled liver surgery to be performed safely for large tumors. Rahimli et al., in their study comparing laparoscopic and robotic surgery for liver colorectal metastases, showed that although patients in the robotic surgery group had larger tumors and more often needed a major resection, robotic surgery had comparable results and was even associated with a higher R0 resection rate [85]. Magistri et al. reported that robotic surgery had comparable R0 resection rates with the laparoscopic approach and that robotic surgery provided safe access to difficult-to-resect segments such as segments I, VII, and VIII, while patients had significantly larger tumors in the robotic surgery group, supporting the superiority of the robotic approach for complex procedures and large tumors [86]. Although it is thought that large tumors may cause high conversion rates and difficulty in obtaining negative surgical margins during minimally invasive surgery, Beard et al. reported lower conversion rates in the robotic surgery group, compared to laparoscopic surgery (5.2% vs 12.2%, respectively), while more patients in the robotic surgery group had large tumors (>5 cm) [77]. In a meta-analysis conducted by Rahimli et al. including comparative studies for robotic and laparoscopic liver surgery, six studies were included in the meta-analysis of tumor size, and it was reported that the tumor size was significantly larger in the robotic group [87]. However, in the results of a survey on the implementation of minimally invasive liver surgery by Zwart et al., a large tumor size (>10 cm) was mentioned as a contraindication to minimally invasive liver surgery by 29% of the participants [88]. These conflicting data make it impossible to reach an evidence-based conclusion about the effect of tumor size on the feasibility of robotic liver surgery.

## 6. Future Outlook

Robotic surgical systems—which were developed to reduce the errors caused by lack of dexterity and human-related factors with the feedback they provide during the implementation of the defined surgical procedures—have been used for nearly 20 years and in liver surgery since 2006 [7]. Thanks to robotic surgical systems, surgeons can perform surgeries in anatomical locations that are difficult to reach with conventional open or laparoscopic surgical systems in a more sensitive, flexible, and controlled manner. Furthermore, robotic surgical systems might prevent fatigue-related surgical errors with their ergonomic design [89,90]. The earlier mentioned advantages of robotic surgery and its acceptable results compared to other surgical approaches have allowed this method to become widespread.

The lack of haptic feedback of the surgical robot is a major challenge of current systems, and this deficiency is only tolerated by the growing experience of surgeons in robotic surgery. However, with the force feedback mechanisms that are to be integrated into the existing system in future versions, learning curves will likely be shortened and complications related to applied tissue tension that may be encountered during the learning phase should be prevented [91].

The current version of the robotic surgical system allows single port surgery with articulating cameras and hand tools, enabling its application in major oncological surgical procedures, causing less postoperative pain, better cosmetic results, and lower hernia rates [92]. Single port liver surgery should limit the abdominal wall trauma even more, especially in case of lesions in the posterosuperior segments, which are somewhat more difficult to reach with open and laparoscopic methods and require larger incisions. In that context, left lateral sectionectomies performed with a robotic single port system can be seen as a benchmark for the future of robotic liver surgery [93].

### 6.1. Training

Robotic systems facilitate surgeons to train their minimally invasive skills before they start performing procedures on patients by allowing virtual reality simulation, training with procedure-specific bio tissue, and on-site proctoring with a second robotic console. A structured training program in robotic pancreatoduodenectomy employing the previously mentioned training methods has been shown to be feasible on a nationwide scale, allowing surgeons to surpass the learning curve without a negative impact on perioperative outcomes [94].

### 6.2. Integrated Imaging

**Figure 2:**
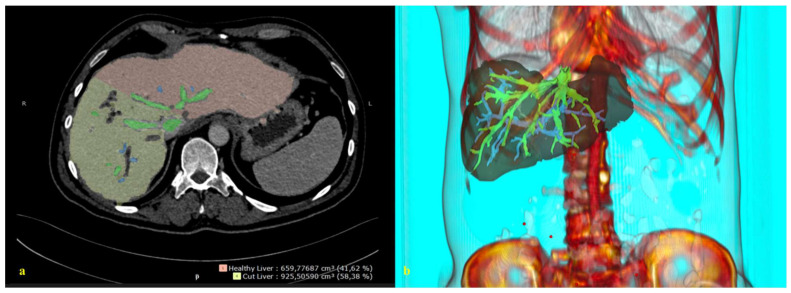
A 3D reconstruction of the radiological image. (**a**) A 2D abdominal computerized tomography scan, axial. (**b**) A 3D reconstruction and image integration of the portal and hepatic venous system of the liver.

By integrating 3D reconstructions of preoperative radiological images into the robotic system, the relationship between the tumor, vascular structures, and biliary anatomy can be revealed, enabling the performance of navigation-based surgeries in daily clinical practice [95] (Figure 2). The data obtained during the preoperative period can be checked by the surgeon during the procedure, facilitating faster and more accurate intraoperative decision-making and possibly improving postoperative outcomes.

In the literature, it is stated that 3D reconstructed images of the liver (detailed vascular and biliary anatomy) can be superimposed on the liver at a ratio of one-to-one to help the surgeon determine the location of the tumor, vascular structures, bile ducts, and transection plane during surgery [96]. This would allow the operations to be performed in a shorter time with less bleeding.

### 6.3. Artificial Intelligence and Machine Learning

Artificial intelligence (AI) is the capacity of a computer system to perform tasks that are normally performed by humans. Machine learning is a subcategory of AI which uses algorithms created by processing data from large datasets, allowing computer systems to learn from this data without specific programming. Machine learning enables real-time observance and direct feedback on surgical performance, improving the ability of repetitive accuracy, and overcoming human limitations such as fatigue and emotional status [97]. Therefore, it is thought that the implementation of AI can further improve perioperative and oncological outcomes, as the surgeons’ decision-making process and actions would be based on up-to-date data.

### 6.4. Mainstream Types of Robots for Liver Surgery

The da Vinci surgical system (Intuitive Surgical, Sunnyvale, CA, USA) is the first robotic surgical system that was developed, and currently, more than 1.2 million surgical procedures are performed annually with this system [98]. Other robotic surgical systems for abdominal surgery which are currently in use or will be in the foreseeable future are: Senhance ALF-X system (TransEnterix, Morrisville, NC, USA); The Revo-i (Meere Company, Yongin, Korea); Micro Hand S, Flex Robotic System (Medrobotics, Raynham, MA, USA); SPORT (Titan medical, Toronto, SD, Canada); Versius (Cambridge Medical Robotics, Cambridgeshire, UK); MiroSurge (Medtronic, Minneapolis, MN, USA); Bitrack System (Rob Surgical Systems SL, Barcelona, Spain); Hintori (Medicaroid, Kobe, Japan); Verb Surgical (Google and Johnson & Johnson); SurgiBot (TransEnterix, Morrisville, NC, USA); Avatera (avateramedical GmbH, Germany); and Hugo RAS (Medtronic, Minneapolis, MN, USA) [98,99]. The robotic surgical systems for which preclinical and clinical studies have been performed for abdominal surgery are Hugo RAS, Senhance, SPORT, Versius and Revo-i. During these preclinical and clinical studies, these systems have been used to perform low anterior resections, abdominoperineal resections, inguinal hernia repairs, cholecystectomies, Nissen fundoplications, splenectomies, enterotomies and pancreaticoduodenectomies [100,101,102,103]. To the best of our knowledge, liver surgery has thus far only been performed using the da Vinci system.

## 7. Conclusions

Minimally invasive liver surgery continues to evolve in parallel with developments in technology and surgical techniques. Robotic liver surgery has been shown to be safe, feasible, and can offer comparable merits over open liver surgery as laparoscopy. Additionally, robotic liver surgery seems to offer a small intraoperative benefit over laparoscopy in technically complex settings in terms of a small decrease in intraoperative blood loss and lower conversion rates.

Although robotic surgical systems have a smoother and wider range of motion compared to conventional laparoscopy, due to the six-axis mobility of their arms and integrated 3D image systems, an important disadvantage is that the current systems do not provide feedback on tissue tension. Another important disadvantage of this technique is the high costs in its current form, while it is predicted that these costs will decrease soon due to the presence of multiple suppliers and the presence of new robotic surgery systems in the market.

It is also foreseen that robotic surgery systems will enable abdominal surgery with navigation in the near future, with wider implementation of imaging modalities. Furthermore, the expectation is that artificial intelligence will increasingly be embraced.

In parallel with the technological developments of robotic surgical systems and the implementation of integrated radiological imaging systems and artificial intelligence technologies, there is no doubt that the robots’ advantages in liver surgery, involving an organ with a very complex arterial, venous and biliary anatomy, will increase.
